# O-GlcNAcylation stabilizes the autophagy-initiating kinase ULK1 by inhibiting chaperone-mediated autophagy upon HPV infection

**DOI:** 10.1016/j.jbc.2022.102341

**Published:** 2022-08-03

**Authors:** Yingxin Shi, Sheng Yan, Guang-Can Shao, Jinglong Wang, Yong-Ping Jian, Bo Liu, Yanqiu Yuan, Ke Qin, Shanshan Nai, Xiahe Huang, Yingchun Wang, Zhenghui Chen, Xing Chen, Meng-Qiu Dong, Yiqun Geng, Zhi-Xiang Xu, Jing Li

**Affiliations:** 1 Beijing Key Laboratory of DNA Damage Response and College of Life Science, Capital Normal University, Beijing, China; 2 National Institute of Biological Sciences, Beijing, China; 3 Qingdao University Medical College Affiliated Hospital, Qingdao, Shandong, China; 4 School of Life Sciences, Henan University, Kaifeng, Henan, China; 5 School of Pharmaceutical Sciences, Sun Yat-sen University, Guangzhou, Guangdong, China; 6 College of Chemistry and Molecular Engineering, Beijing National Laboratory for Molecular Sciences, Peking-Tsinghua Center for Life Sciences, Synthetic and Functional Biomolecules Center, and Key Laboratory of Bioorganic Chemistry and Molecular Engineering of Ministry of Education, Peking University, Beijing, China; 7 State Key Laboratory of Molecular Developmental Biology, Institute of Genetics and Developmental Biology, Chinese Academy of Sciences, Beijing, China; 8 State Key Laboratory of Bioactive Substance and Function of Natural Medicines, Institute of Materia Medica, Chinese Academy of Medical Sciences and Peking Union Medical College, Beijing, China; 9 State Key Laboratory of Natural and Biomimetic Drugs, Peking University, Beijing, China

**Keywords:** O-GlcNAc, ULK1, HPV, HNSCC, macroautophagy, chaperone-mediated autophagy, 5S-G, acetyl-5S-GlcNAc, AMPK, AMP-activated protein kinase, CQ, chloroquine, ETD, electron transfer dissociation, Glu, glucose, HNSCC, head and neck squamous cell carcinoma, HPV, human papillomavirus, HSC70, heat shock cognate 70 kDa protein, IP, immunoprecipitation, MS, mass spectrometry, mTOR, mammalian target of rapamycin, O-GlcNAc, O-linked β-*N*-acetylglucosamine, OGA, O-GlcNAcase, OGT, O-GlcNAc transferase, OGTi, OGT inhibitor, PPase, phosphatase, PTM, post-translational modification, TMG, Thiamet-G, ULK1, Unc-51-like kinase 1

## Abstract

Human papillomaviruses (HPVs) cause a subset of head and neck squamous cell carcinomas (HNSCCs). Previously, we demonstrated that HPV16 oncogene *E6* or *E6/E7* transduction increases the abundance of O-linked β-*N*-acetylglucosamine (O-GlcNAc) transferase (OGT), but OGT substrates affected by this increase are unclear. Here, we focus on the effects of O-GlcNAcylation on HPV-positive HNSCCs. We found that upon HPV infection, Unc-51-like kinase 1 (ULK1), an autophagy-initiating kinase, is hyper-O-GlcNAcylated, stabilized, and linked with autophagy elevation. Through mass spectrometry, we identified that ULK1 is O-GlcNAcylated at Ser409, which is distinct from the previously reported Thr635/Thr754 sites. It has been demonstrated that PKCα mediates phosphorylation of ULK1 at Ser423, which attenuates its stability by shunting ULK1 to the chaperone-mediated autophagy (CMA) pathway. Using biochemical assays, we demonstrate that ULK1 Ser409Ser410 O-GlcNAcylation antagonizes its phosphorylation at Ser423. Moreover, mutations of Ser409A and its neighboring site Ser410A (2A) render ULK1 less stable by promoting interaction with the CMA chaperone HSC70 (heat shock cognate 70 kDa protein). Furthermore, ULK1-2A mutants attenuate the association of ULK1 with STX17, which is vital for the fusion between autophagosomes and lysosomes. Analysis of The Cancer Genome Atlas (TCGA) database reveals that ULK1 is upregulated in HPV-positive HNSCCs, and its level positively correlates with HNSCC patient survival. Overall, our work demonstrates that O-GlcNAcylation of ULK1 is altered in response to environmental changes. O-GlcNAcylation of ULK1 at Ser409 and perhaps Ser410 stabilizes ULK1, which might underlie the molecular mechanism of HPV-positive HNSCC patient survival.

O-linked β-*N*-acetylglucosamine (O-GlcNAc) involves the addition of the O-GlcNAc moiety to protein Ser/Thr residues (1, 2). This quintessential post-translational modification (PTM) is catalyzed by the sole O-GlcNAc transferase (OGT) in humans and reversed by the mere O-GlcNAcase (OGA). Traditionally, O-GlcNAcylation identification has been hindered by its low stoichiometry, extreme lability, and lack of gel retardation during electrophoresis (1, 2). However, with the development of click chemistry to label O-GlcNAcylated proteins, together with great strides in mass spectrometry (MS) technologies, an array of quantitative MS analyses has been performed and provides a path to study the biological perspectives of O-GlcNAc (3, 4, 5, 6).

As a nutrient-sensing PTM, O-GlcNAc undergoes dynamic changes under different stimuli. Glucose (Glu) deprivation and fasting, for instance, could promote protein O-GlcNAcylation (7, 8, 9). Upon viral infection, such as with human papillomavirus (HPV), O-GlcNAcylation is also enhanced (10). Currently, about 5000 proteins are estimated to be O-GlcNAcylated in humans and mice (www.oglcnac.org) (11). O-GlcNAcylation is known to crosstalk with other PTMs, for instance, by phosphorylation and ubiquitination (1). O-GlcNAc sometimes antagonizes phosphorylation and sometimes promotes phosphorylation, depending on the protein being studied. Like O-GlcNAcylation, phosphorylation also results from different stimuli in response to a variety of signaling pathways and alters protein stability and function. Unlike O-GlcNAcylation, phosphorylation is mediated by about 600 kinases, so activation or inactivation of different kinases could modulate distinct modification sites on the same protein. In stark contrast, with only one OGT present in humans, do the OGT substrates alter O-GlcNAc sites or O-GlcNAc levels under environmental stresses? If so, would different biological consequences ensue?

In this work, we focused on the autophagy pathway, as it is also sensitive to nutrient status. Autophagy, or macroautophagy, is a survival mechanism; it refers to the process of engulfing cytoplasmic components into double-membrane autophagosomes and subsequent delivery for lysosomal degradation, allowing the cells to survive by salvaging the digested cellular components (12). Its initiation relies largely on the crosstalk and feedback among the mammalian target of rapamycin (mTOR)–AMP-activated protein kinase (AMPK)–Unc-51-like kinase 1 (ULK1) trio, with AMPK sensing energy, mTOR detecting nutrients, and ULK1 initiating autophagy (13). Upon activation, ULK1 modulates the nucleation and membrane fusion of Atg9 (autophagy-related 9) and COPII (coat protein II) vesicles to enable autophagosome biogenesis (12). This process is also facilitated by class III PI3K complex I.

As a vital kinase for autophagy, ULK1 is subject to a range of PTMs. For instance, during Glu starvation, AMPK or mTOR could directly phosphorylate ULK1 at Ser317 and Ser777 and activate ULK1 (14, 15, 16). When nutrients are sufficient, mTOR phosphorylates ULK1 at Ser757 and inhibits its activity by dissociating ULK1 from AMPK (14, 15, 16). During amino acid deprivation, ULK1 undergoes dephosphorylation at Ser638 and Ser758 and dissociates from AMPK, and the S758A mutant initiates autophagy at a faster pace (17). During starvation-induced liver autophagy, ULK1 is O-GlcNAcylated at Thr635 and Thr754, which enhances ULK1–AMPK interaction, promotes phosphorylation at Ser555 and Ser638, and activates ULK1 (18). Furthermore, Thr754 O-GlcNAcylation is key for ULK1–ATG14L binding. ATG14L is thus activated and mediates downstream events, including VPS34 activation, phosphatidylinositol-(3)-phosphate production, and phagophore formation (19).

Recently, ULK1 has also been demonstrated to be phosphorylated by PKCα at Ser423 (20). On one hand, this PTM enhances degradation *via* interaction with HSC70 (heat shock cognate 70 kDa protein) and the chaperone-mediated autophagy (CMA) pathway. On the other hand, pSer423 also attenuates ULK1–STX17 binding, thus decreasing fusion events between autophagosomes and lysosomes (20). This multilayered regulation of ULK1 epitomizes these intricate biological signaling networks.

Here, we identified new ULK1 O-GlcNAc sites under high O-GlcNAcylation. Upon HPV infection, O-GlcNAc levels were elevated. By monitoring ULK1 O-GlcNAc levels and MS analysis, we identified new O-GlcNAc sites. *Via* biochemical and cellular assays, we found that O-GlcNAcylation at Ser409 and its neighboring Ser410 antagonizes pSer423, leading to ULK1 stabilization and enhanced autophagy. We propose that the dynamic glycosylation cycle could alter O-GlcNAcylation sites to respond to disparate environmental cues, so that distinct biological pathways are regulated to result in different biological consequences.

## Results

### HPV infection increases ULK1 O-GlcNAcylation

Previously, we showed that upon HPV infection, O-GlcNAcylation is increased (10). We wondered whether this elevation would have an effect on the autophagy pathway, because autophagy is sensitive to nutrient status (21), and O-GlcNAc is a nutrient-sensitive PTM (2). We selected ULK1, because ULK1 not only has been shown to be O-GlcNAcylated at Thr635 and Thr754 (18) but also initiates autophagy.

**Figure 1:**
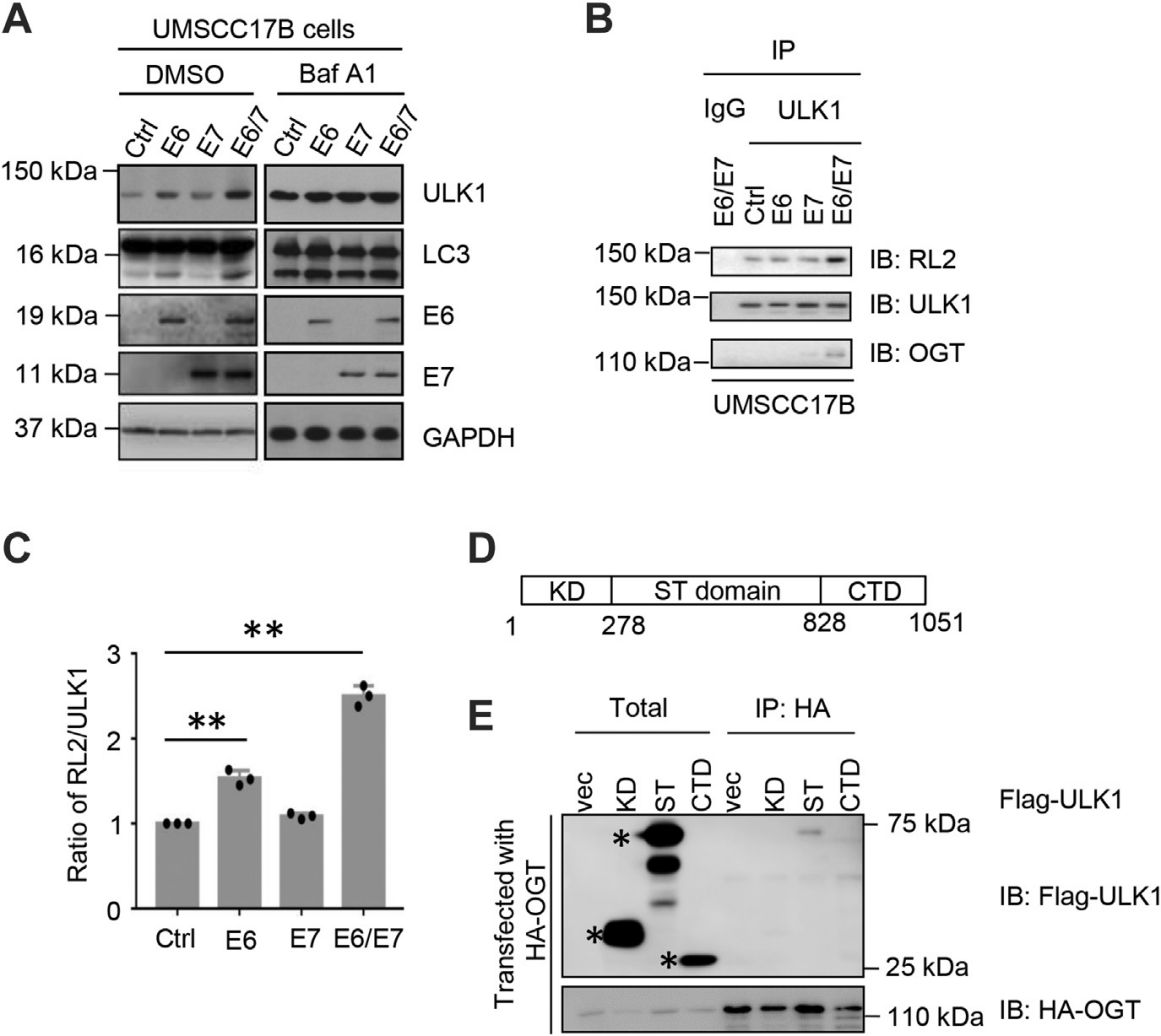
**HPV infection elevates O-GlcNAc of ULK1, ULK1 protein abundance, and autophagic flux.***A*, to mimic HPV infection, human HNSCC UMSCC17B cells were infected with lentiviruses expressing HPV16 E6, E7, E6/E7, or empty vectors and selected with 1 μg/ml puromycin for 2 weeks. The surviving colonies were combined. Cells were treated with 200 nM bafilomycin A1 (Baf A1) or DMSO for 16 h. Whole-cell extracts were isolated and immunoblotted with the antibodies indicated. GAPDH was detected as loading control. *B*, cell lysates collected from UMSCC17B/Ctrl, E6, E7, and E6/E7 cells were subject to immunoprecipitation and immunoblotting as indicated. *C*, quantitation of (*B*), showing the ratio of the signal of RL2 normalized to ULK1. ∗∗ indicates significant differences as determined by one-way ANOVA (*p* < 0.01). *D*, schematic showing the kinase domain (KD), Ser/Thr-rich domain (ST), and the C-terminal domain (CTD) of ULK1. *E*, HeLa cells were transfected with HA-OGT and disparate constructs of ULK1 fragments and then subjected to immunoprecipitation and immunoblotting with antibodies indicated. The *asterisk* indicates the purified protein of interest. HA, hemagglutinin; HNSCC, head and neck squamous cell carcinoma; HPV, human papillomavirus; O-GlcNAc, O-linked β-*N*-acetylglucosamine; OGT, O-GlcNAc transferase; ULK1, Unc-51-like kinase 1.

As HPV infection could lead to head and neck squamous cell carcinomas (HNSCCs), we used human HNSCC UMSCC17B cells. They were infected with lentiviruses expressing HPV16 E6, E7, and E6/E7, and LC3 was examined to determine autophagic levels. As shown in Figure 1*A*, autophagy levels increased in HPV16 E6 and E6/7-infected cells, concomitant with the increase in ULK1. We also used bafilomycin A1, an inhibitor for autophagosome–lysosome fusion, and observed the accumulated autophagy marker LC3-II (Fig. 1*A*).

Because we previously demonstrated that HPV infection increases O-GlcNAcylation levels in cervical neoplasms (10), and ULK1—the initiating kinase for autophagy—has been shown to be O-GlcNAcylated (18), we sought to assess whether the aforementioned phenotype is caused by elevated ULK1 O-GlcNAcylation. UMSCC17B cells were again transfected with HPV16 E6, E7, and E6/E7 to mimic HPV infection. In the HPV16 E6/E7-infected cells, there is a significant increase of OGT binding with ULK1 as well as ULK1 O-GlcNAc signals as detected by RL2 antibodies (Fig. 1, *B* and *C*).

As ULK1 is O-GlcNAcylated under nutrient starvation, we wondered whether it could also be glycosylated upon high O-GlcNAc. To this end, we first strove to identify which domain of ULK1 interacts with OGT. As ULK1 comprises a kinase domain, a Ser/Thr-rich domain domain, and a C-terminal domain (22), we made constructs accordingly (Fig. 1*D*). These plasmids were cotransfected into cells with hemagglutinin-OGT, and the Ser/Thr-rich domain domain was responsible for the interaction, suggesting that the potential O-GlcNAc sites might be in this region (Fig. 1*E*).

Previous reports have linked O-GlcNAc of ULK1 with its kinase activity (18), but we observed an increase in ULK1 protein abundance upon HPV infection. Thus, we speculated that there might be other O-GlcNAc sites on ULK1 that modulate its protein levels.

### ULK1 is O-GlcNAcylated at Ser409 upon increased O-GlcNAcylation

**Figure 2:**
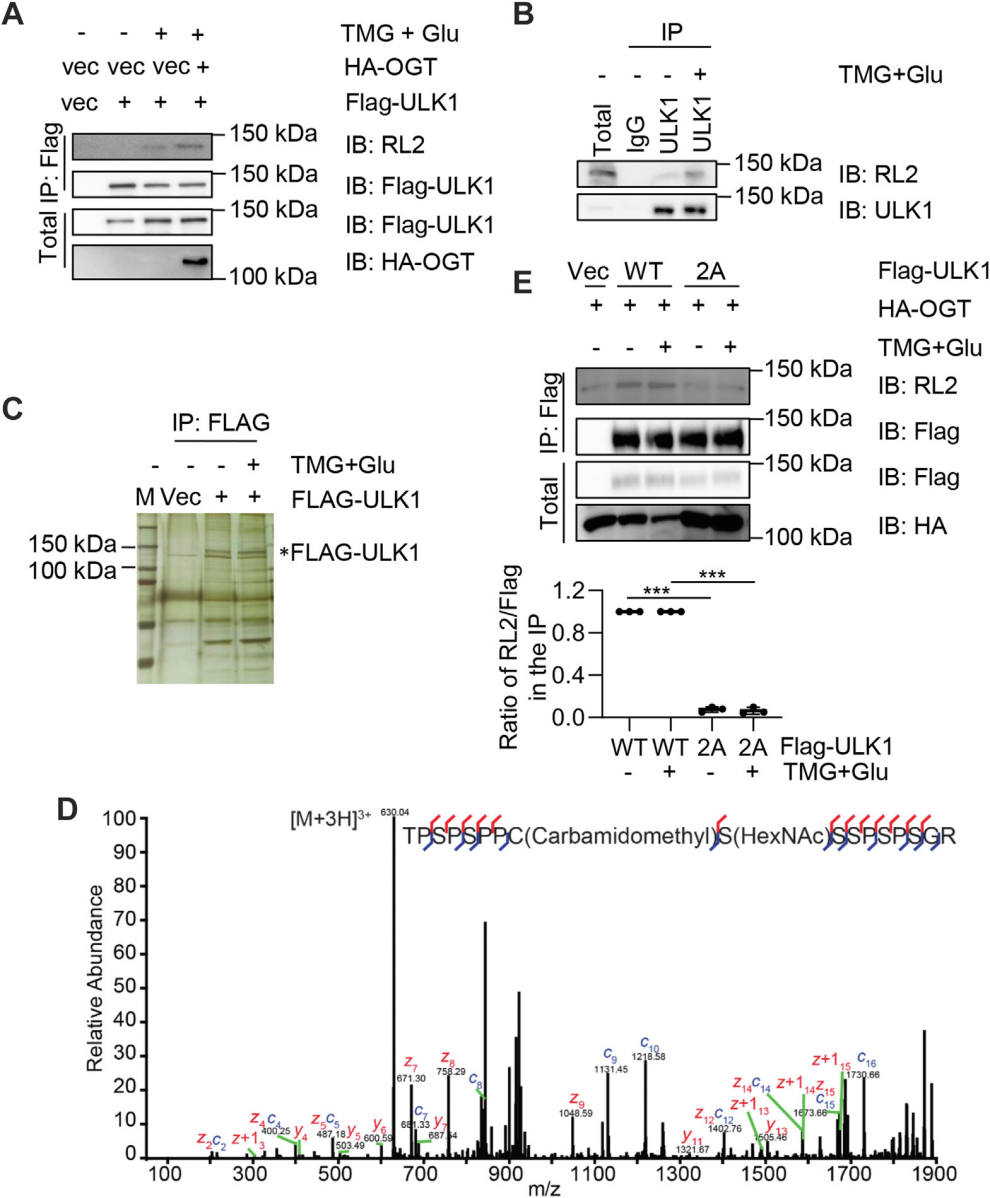
**Upon elevated O-GlcNAcylation, ULK1 is O-GlcNAcylated at Ser409.***A*, HeLa cells were transfected with HA-OGT and FLAG-ULK1 plasmids and then treated with 30 mM TMG plus glucose (TMG + Glu) or left untreated. Cells were then subjected to immunoprecipitation and immunoblotting with indicated antibodies. *B*, HeLa cells were treated with TMG + Glu or left untreated, immunoprecipitated with anti-ULK1 antibodies, and immunoprecipitated with anti-ULK1 or RL2 antibodies. *C*, HeLa cells were transfected with FLAG-ULK1 plasmids and enriched for O-GlcNAc by TMG + Glu treatment. Cell lysates were immunoprecipitated with anti-FLAG M2 agarose and then subjected to silver staining. The *asterisk* denotes FLAG-ULK1. *D*, ETD MS/MS spectrum of the O-GlcNAcylated peptide TPSPSPPC(carbamidomethyl)S(HexNAc)SSPSPSGR on ULK1. The matched fragment ions are shown in *red* and *blue*. *E*, HeLa cells were transfected with HA-OGT together with ULK1-S409AS410A (2A). Cells were then treated with TMG + Glu, and the resultant lysates were collected and subjected to immunoprecipitation and immunoblotting. ∗∗∗ indicates significant differences as determined by one-way ANOVA (*p* < 0.001). ETD, electron transfer dissociation; HA, hemagglutinin; O-GlcNAc, O-linked β-*N*-acetylglucosamine; OGT, O-GlcNAc transferase; TMG, Thiamet-G; ULK1, Unc-51-like kinase 1.

We next examined whether ULK1 is glycosylated upon increased O-GlcNAcylation. Figure 2*A* shows the elevated ULK1 O-GlcNAcylation when cells were cultured in Thiamet-G (TMG) plus Glu, as described previously (23) and in the Experimental procedures section. When endogenous ULK1 was examined, TMG plus Glu also enhanced O-GlcNAc levels (Fig. 2*B*).

We then identified the glycosylation sites upon increased O-GlcNAcylation. Cells were transfected with FLAG-ULK1 and treated with TMG plus Glu, and silver staining showed a specific band corresponding to FLAG-ULK1 (Fig. 2*C*). The immunoprecipitates were then subject to electron transfer dissociation (ETD) MS as described in the Experimental procedures section. A single peptide was identified, and Ser409 was determined to the O-GlcNAcylated sites (Fig. 2*D*), in contrast to the Thr635 and Thr754 sites as previously reported (18, 19).

Because we identified Ser409 from the MS analysis, we decided to mutate both Ser409 and its neighboring Ser410 residues. This is a common approach in the field, as was first published for the estrogen receptor beta (24). We then generated glycosylation-deficient Ser409Ala Ser410Ala (2A) mutants. Upon transfection, 2A attenuated O-GlcNAc signals substantially (Fig. 2*E*), indicating that they are the major glycosylation sites under these conditions.

### ULK1-2A increases binding with HSC70 in the CMA pathway

We first examined whether 2A mutation affects ULK1 ubiquitination levels, as the ubiquitin-proteasome pathway usually regulates protein abundance, and O-GlcNAc has been shown to crosstalk with ubiquitination in many proteomic studies (25). To our surprise, the O-GlcNAc-deficient 2A mutant does not affect ULK1 ubiquitination (data not shown).

**Figure 3:**
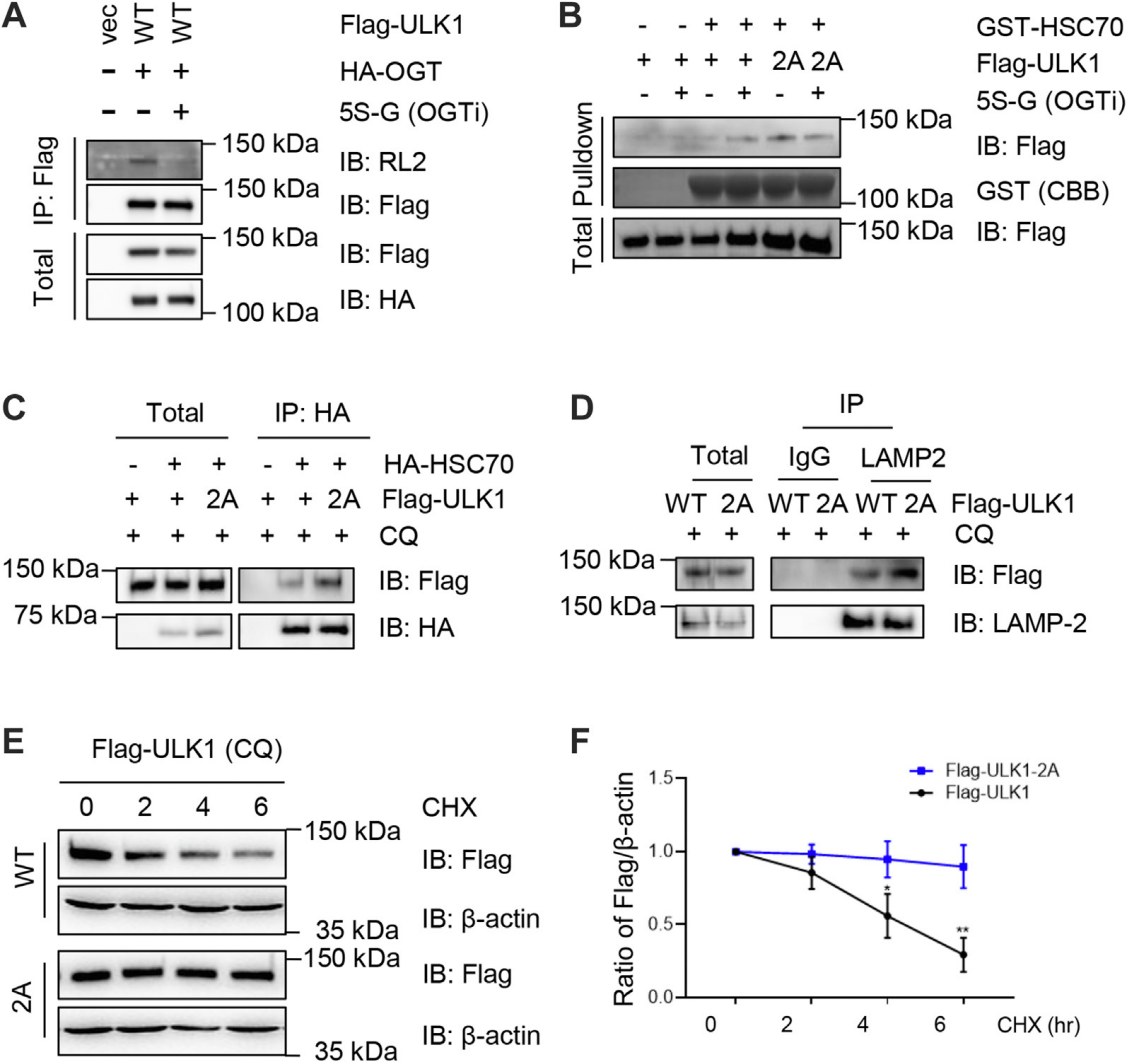
**ULK1-2A enhances its interaction with HSC70, a key protein in the chaperone-mediated autophagy (CMA) pathway.***A*, cells were transfected with FLAG-ULK1 and HA-OGT plasmids, treated with 5S-G (OGT inhibitor), or left untreated. The anti-FLAG immunoprecipitates were immunoblotted with the antibodies indicated, suggesting that 5S-G could efficiently inhibit ULK1 O-GlcNAcylation. *B*, cells were transfected with ULK1-WT or ULK1-2A plasmids, treated with 5S-G (OGT inhibitor), or left untreated. The cellular lysates were then incubated with recombinant GST-HSC70 proteins, and GST pull-down assays were carried out. CBB refers to Coomassie brilliant blue, which indicates the total of GST-HSC70 proteins. *C*, cells were transfected with ULK1-WT or ULK1-2A plasmids together with HA-HSC70, then incubated with chloroquine (CQ) (100 nM for 3 h). *D*, cells were transfected with FLAG-ULK1-WT or FLAG-ULK1-2A plasmids and treated with CQ. The cell lysates were immunoprecipitated with anti-LAMP-2 antibodies. *E* and *F*, cells were transfected with FLAG-ULK1-WT or FLAG-ULK1-2A plasmids and treated with CQ and cycloheximide (CHX). While CHX induces protein level decreases in WT-ULK1, the 2A mutant displayed stable protein abundance, suggesting that the majority of the 2A mutant was degraded *via* the CMA pathway. Quantitation in (*F*) indicates the ratio of FLAG-ULK1 normalized to β-actin. A two-way ANOVA test was used for statistical analysis. ∗ indicates *p* < 0.05, ∗∗ indicates *p* < 0.01. 5S-G, acetyl-5S-GlcNAc; GST, glutathione-*S*-transferase; HA, hemagglutinin; HSC70, heat shock cognate 70 kDa protein; OGT, O-GlcNAc transferase; ULK1, Unc-51-like kinase 1.

We reviewed the literature to find other factors that regulate ULK1 protein levels. It has been reported that ULK1 is phosphorylated by PKCα at Ser423 to promote the binding affinity between ULK1 and Hsc70, the molecular chaperone for the CMA pathway (20). Indeed, ULK1-2A elevated its binding with Hsc70 in a glutathione-*S*-transferase pull-down assay (Fig. 3*B*). The interaction also increased when we treated the cells with the OGT inhibitor (OGTi) acetyl-5S-GlcNAc (5S-G), suggesting that the alteration was caused by O-GlcNAc modification (Fig. 3*B*). To verify that 5S-G (OGTi) indeed decreased ULK1 O-GlcNAcylation levels, cells were transfected with FLAG-ULK1 and hemagglutinin-OGT and treated or untreated with 5S-G (Fig. 3*A*). Chemical utilization nearly abolished ULK1 O-GlcNAcylation (Fig. 3*A*).

We also examined the interaction between ULK1-WT and ULK1-2A with HSC70 in cells. Upon incubation with the lysosome inhibitor chloroquine (CQ), ULK1-2A enhanced binding with Hsc70 (Fig. 3*C*). Moreover, we examined the interaction between ULK1-2A and Lamp2, a protein localizing on the lysosome membrane. As shown in Figure 3*D*, an increase between ULK1-2A and Lamp2 was observed, suggesting that more ULK1-2A proteins are subject to the CMA pathway.

We then sought to examine whether ULK1-2A is degraded in the lysosome by using CQ to inhibit the lysosome. As shown in Figure 3*E*, cells were pretreated with cycloheximide for 3 h and then incubated together with CQ. While this treatment induced a marked decrease in ULK1-WT protein stability, 2A proteins remained stable (Fig. 3*F*). These results suggest that ULK1-WT could be degraded *via* both the ubiquitin-proteasome pathway and the CMA pathway, and that ULK1-2A is mainly degraded *via* the lysosome-mediated CMA pathway.

### ULK1-2A reduces binding with STX17 and subsequent fusion of autophagosomes and lysosomes

**Figure 4:**
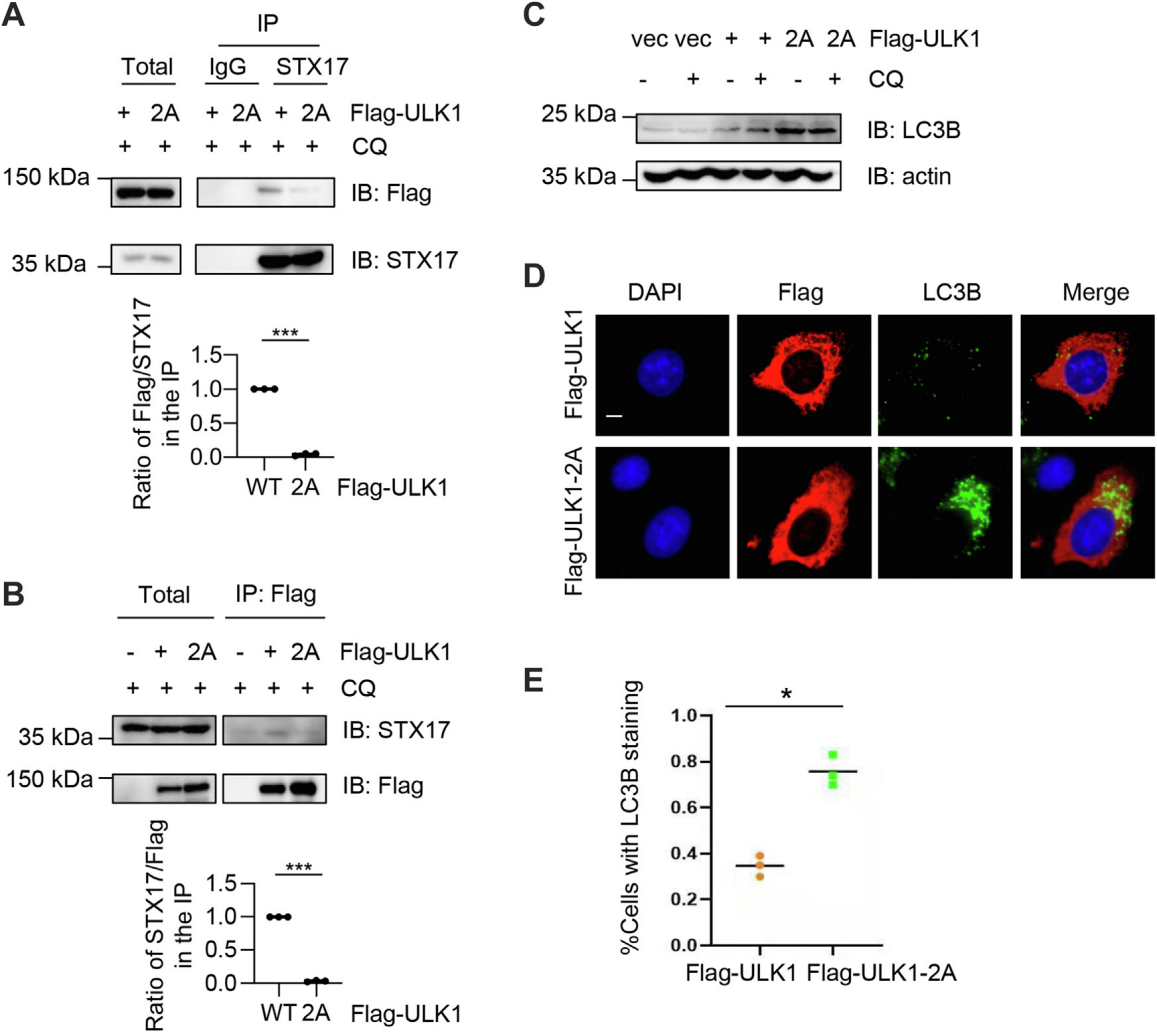
**ULK1-2A limits ULK1–STX17 interaction, resulting in defective macroautophagy.***A* and *B*, ULK1-2A decreased interaction with STX17. Cells were transfected with FLAG-ULK1-WT or FLAG-ULK1-2A and then treated with CQ. Lysates were subjected to immunoprecipitation and immunoblotting as indicated. Quantitation indicates the ratio of the FLAG signal normalized to STX17 in the IP (*A*) or the ratio of the STX17 signal normalized to FLAG in the IP (*B*). ∗∗∗ indicates *p* < 0.001. *C*, cells were transfected with FLAG-ULK1-WT or FLAG-ULK1-2A and treated with CQ. Autophagic levels were monitored with LC3B antibodies. *D*, cells were transfected with FLAG-ULK1-WT or FLAG-ULK1-2A plasmids and then stained with anti-LC3B antibodies and DAPI. The scale bar represents 10 μm. *E*, quantitation of LC3B-positive cells. WT cells were normalized as 1. ∗ indicates significant differences as determined by Student’s *t* test (*p* < 0.05). CQ, chloroquine; DAPI, 4′,6-diamidino-2-phenylindole; IP, immunoprecipitation; ULK1, Unc-51-like kinase 1.

It has been reported that unphosphorylated ULK1 recruits STX17 to promote autophagosomes and the subsequent autophagosome–lysosome fusion (20). We hypothesized that O-GlcNAc might have an adverse effect. We attempted to determine the binding affinity between ULK1-2A and STX17. Indeed, ULK1-2A impaired ULK1–STX17 association, as determined by coimmunoprecipitation (IP) assays (Fig. 4, *A* and *B*), suggesting that 2A is defective in STX17 recruitment.

We then examined the autophagosome and lysosome fusion by LC3B. Cells were transfected with FLAG-ULK1-WT or FLAG-ULK1-2A and then treated with CQ. As shown in Figure 4*C*, CQ treatment elevated LC3B levels in FLAG-ULK1-WT cells, but remained at a stably high level in FLAG-ULK1-2A cells. We also monitored LC3B staining by immunofluorescence (Fig. 4, *D* and *E*). In FLAG-ULK1-2A transfected cells, a marked increase of LC3B-stained cells was discernable, suggesting that more cells are defective in the fusion process.

### ULK1-Ser409 O-GlcNAcylation antagonizes Ser423 phosphorylation

**Figure 5:**
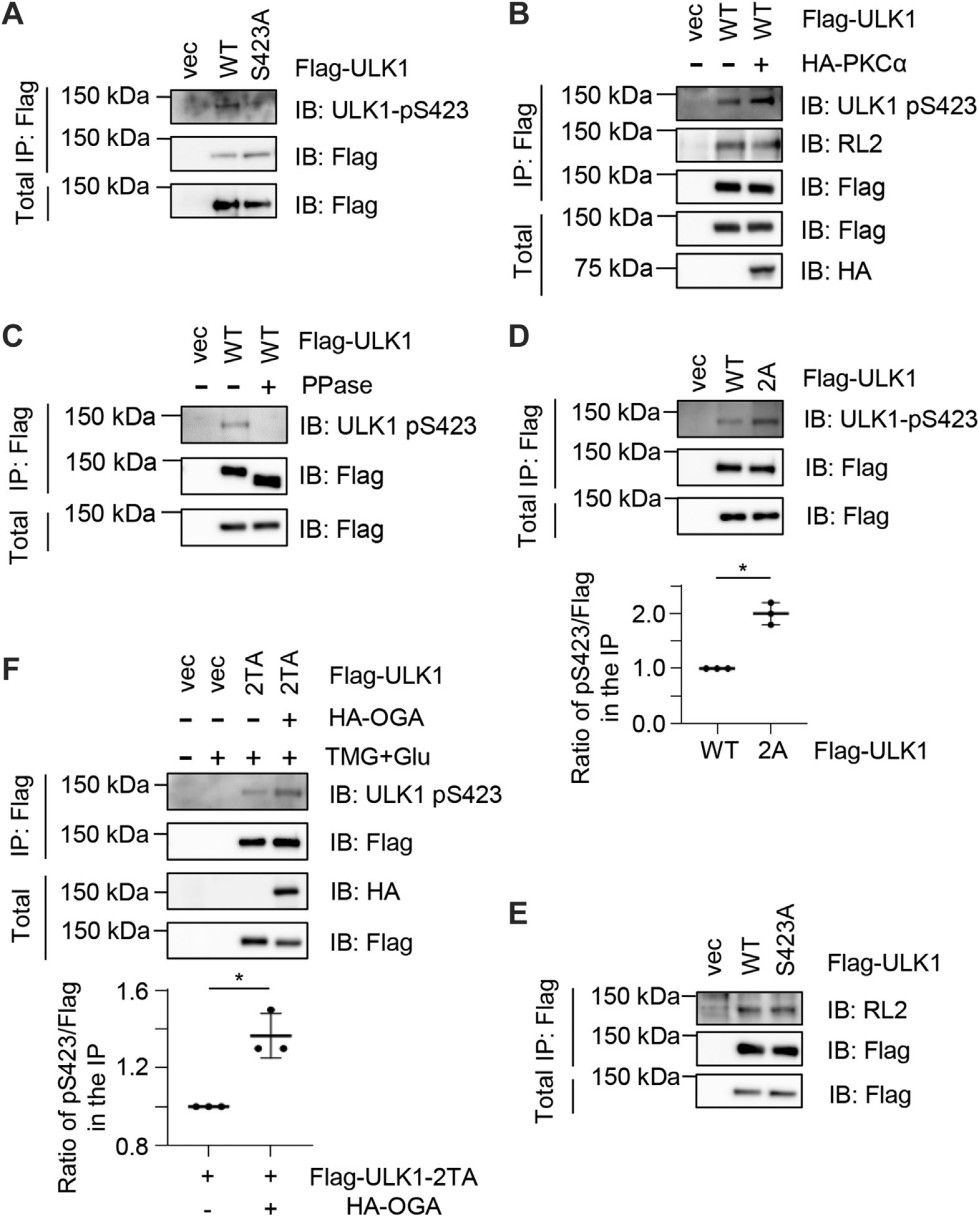
**ULK1 O-GlcNAcylation antagonizes pS423.***A*, a rabbit anti-pS423 antibody was generated. Cells were transfected with FLAG-ULK1-WT or FLAG-ULK1-S423A, and the lysates were immunoprecipitated and immunoblotted with the antibodies indicated. *B*, cells were transfected with PKCα plasmids, and the pS423 antibody increases were concomitant with PKCα expression, suggestive of the specificity of the antibody. *C*, cells were transfected with FLAG-ULK1 plasmids. The anti-FLAG immunoprecipitates were subject to λ phosphatase (PPase) treatment or left untreated. They were then subjected to immunoblotting with the indicated antibodies. The decrease of gel mobility in the anti-FLAG immunoblots suggests that PPase treatment efficiently reduced ULK1 phosphorylation. *D* and *E*, cells were transfected with FLAG-ULK1-WT or FLAG-ULK1-2A (*D*), or WT and -S423A (*E*), and then the lysates were immunoprecipitated and immunoblotted with the antibodies indicated. ULK1 O-GlcNAcylation antagonizes pS423, whereas S423 phosphorylation has no effect on O-GlcNAcylation, suggesting that pS423 is downstream of O-GlcNAcylation. Quantitation in *E* indicates the ratio of the pS423 signal normalized to FLAG in the IP. *F*, cells were transfected with ULK1-WT or -T635AT754A (2TA) mutants and then treated as indicated. 2TA displayed higher pS423 levels upon HA-OGA overexpression. Quantitation indicates the ratio of the pS423 signal normalized to FLAG in the IP. ∗ in *D* and *F* indicates significant differences as determined by Student’s *t* test (*p* < 0.05). HA, hemagglutinin; IP, immunoprecipitation; OGA, O-GlcNAcase; ULK1, Unc-51-like kinase 1.

As pSer423 mediates the binding between ULK1 and STX17 or Hsc70, we wondered whether O-GlcNAc could crosstalk with pSer423. We first generated a phospho-specific antibody (Fig. 5*A*). To verify the specificity of the phosphor-antibody, we performed three assays. First, when this antibody was used to IP FLAG-ULK1-WT or FLAG-ULK1-Ser423Ala lysates, a specific band was observed in the WT lysates but not in the S423A lysates (Fig. 5*A*). Second, when cells were cotransfected with ULK1 and PKCα plasmids, the antibody increased its signal in the PKCα-transfected lysates (Fig. 5*B*), suggesting it is sensitive to PKCα kinase activity. Last, we used the λ phosphatase (PPase) assay (Fig. 5*C*). Cells were transfected with FLAG-ULK1, and the anti-FLAG immunoprecipitates were incubated with PPase or not. As Figure 5*C* shows, PPase incubation significantly decreased pS423 levels. The pronounced difference in gel shift in the anti-FLAG immunoblots also indicates the efficiency of our PPase treatment. Taken together, the pSer423 antibody shows strong specificity.

We then transfected cells with FLAG-ULK1-WT or FLAG-ULK1-2A plasmids and applied the pSer423 antibody (Fig. 5*D*). An increase in phosphorylation is discernable, suggesting that O-GlcNAc antagonizes phosphorylation. We also wondered whether the reverse was true and tested by monitoring O-GlcNAc levels in the S423A mutant (Fig. 5*E*). However, S423A shows no alteration of O-GlcNAcylation, as detected by RL2 antibodies.

ULK1 was reported to be O-GlcNAcylated at Thr635 and Thr754 (18). Although these two sites were not detected in our MS data, we tried to exclude their potential effect. We generated ULK1-T635AT754A (2TA) mutants and examined phosphorylation when OGA was overexpressed (Fig. 5*F*). OGA overproduction increased pS423 levels. In summary, ULK1-Ser409Ser410 O-GlcNAc could act upstream of pSer423 and display an inhibition effect.

### ULK1 expression correlates with HNSCC

HPV infection can lead to HNSCC. HNSCC is intrinsically of heterogeneous nature, and improvement of 5-year survival rates has been achieved in only a few subtypes (26). Growing numbers of cases of HNSCC have been diagnosed in the oropharynx, etiologically associated with high-risk HPV-16 and HPV-18 (27), and HPV-positive HNSCCs have been shown to have a higher disease-free survival rate (28).

**Figure 6:**
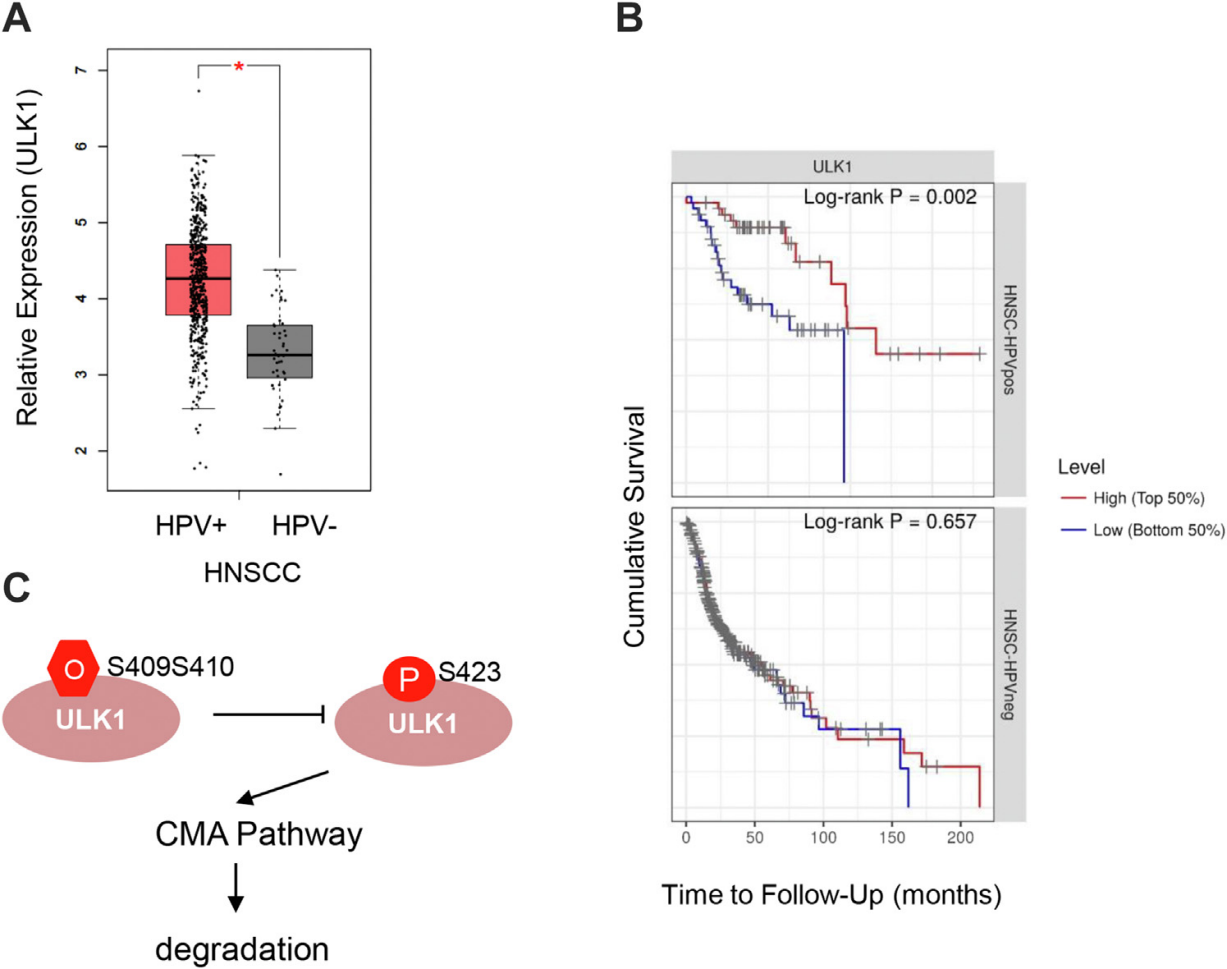
**TCGA database suggests that ULK1 is overexpressed in HPV-positive HNSCC.***A*, ULK1 mRNA levels in HPV-positive and HPV-negative HNSCC samples from TCGA database. *B*, Kaplan–Maier survival curves of HPV-positive and HPV-negative HNSCC patients with high or low ULK1 expression levels (https://cistrome.shinyapps.io/timer/). *p* Value was calculated with the Chi-square test. *C*, model showing that high O-GlcNAc stabilizes ULK1 by inhibiting CMA, thus elevating autophagic flux to promote HPV-positive HNSCC patient survival. CMA, chaperone-mediated autophagy; HNSCC, head and neck squamous cell carcinoma; HPV, human papillomavirus; O-GlcNAc, O-linked β-*N*-acetylglucosamine; TCGA, The Cancer Genome Atlas; ULK1, Unc-51-like kinase 1.

We wondered whether the biochemical and cellular effects we observed could have a physiological role. We first scanned ULK1 expression status across The Cancer Genome Atlas database *via* the cBioPortal. In HNSCC, *ULK1* gene expression levels are elevated in HPV-positive samples, compared with HPV-negative samples (Fig. 6*A*). Importantly, high *ULK1* expression levels correlate with a better prognosis in the HPV-positive HNSCCs (Fig. 1*B*). As ULK1 is the autophagy initiating kinase, these results suggest that autophagy, known to engulf intracellular microbes upon infection (29), could be playing a protective role by engulfing the invading HPV particles, thus protecting patients from HNSCC.

## Discussion

In this report, we identified Ser409 as the O-GlcNAcylation site of ULK1 upon HPV infection, showcasing the dynamics of O-GlcNAc in response to environmental stimuli. This glycosylation event antagonizes pSer423, shunts ULK1 away from CMA, stabilizes ULK1 protein levels, and might be beneficial for HPV-positive HNSCC patients.

The newly identified Ser409 O-GlcNAc site is not conserved in ULK2, but the Ser423 phosphorylation site is. Both ULK1 and ULK2 were identified as human homologs of *Caenorhabditis elegans* Unc-51 (30, 31). As ULK1-deficient animals only display delayed maturation of reticulocytes (32), ULK2 has always been assumed to play a partially redundant role. Perhaps our data highlight minor differences between the two kinases, with ULK1 harboring more PTMs responding to various stresses.

Intriguingly, neither our higher-energy collisional dissociation nor ETD MS revealed Thr635 or Thr754 glycosylated peptides, suggesting that, at least under our experimental settings, Ser409 is the major O-GlcNAc site. It is difficult to delineate the relationship between Ser409 and Thr635Thr754 at this moment. It could be that ULK1 O-GlcNAc occurs at Thr635Thr754 during starvation (18) and at Ser409 under our experimental settings. Another scenario is that Thr635Thr754 still occurs when HPV invades but at a low amount that escapes the detection of MS. A third possibility is that ULK1 is O-GlcNAcylated at Thr635Thr754 during autophagy initiation and at Ser409 during autolysosome formation. A definite characterization of the O-GlcNAc of ULK1 may be reached with quantitative MS in the future.

Several lines of evidence suggest that autophagy downregulation is detrimental for HNSCC survival. In oral cavity HNSCC, impaired autophagy, as evidenced by high cytoplasmic p62 levels, is associated with reduced overall survival (33). Upon HPV-16 infection, the virion activates the PI3K/Akt/mTOR pathway to inhibit autophagy and enhance HPV infection rate (34). Both molecular or chemical inhibition of autophagy elevate HPV infectivity (34, 35). Our study reveals that ULK1 O-GlcNAcylation at Ser409Ser410 promotes both ULK1 stability and autophagosome–lysosome fusion, which could promote HNSCC survival by enhancing autophagy.

The relationship between autophagy and cancer is murky at best. Studies in HNSCC as well as melanoma both point to an autophagy paradox where early stage tumorigenesis dampens autophagy, whereas later-stage cancer cells rely on autophagy for better survival (26, 36). Thus, autophagy is a double-edged sword for killing cancer cells. Our study partially addresses the molecular underpinning of why HPV-positive oropharyngeal cancer patients have a higher survival rate than HPV-negative patients. Furthermore, ULK1 protein levels may serve as a prognostic biomarker.

O-GlcNAcylation has been closely linked with autophagy. On one hand, O-GlcNAc may inhibit autophagy. In *C. elegans*, loss of *ogt-1* (homolog of human OGT) partly elevates autophagy, thus alleviating proteotoxicity associated with neurodegenerative diseases (37). Congruently, abrogation of O-GlcNAcylation sites of SNAP-29 promotes autophagic flux by enhancing fusion between autophagosomes and endosomes/lysosomes in worms as well as human cells (38). On the other hand, paradoxically, nutrient deprivation as well as fasting may promote O-GlcNAcylation. In *Drosophila*, O-GlcNAcylation levels as well as OGT abundance increase during starvation (39). The same is true for human neuronal cells (7) and cancer cells (9), probably because of OGT upregulation when Glu is scarce (8). Recently, it has been shown that direct chemical inhibition of OGA stimulates autophagy in human, mouse, and rat neuronal cells in an mTOR-independent pathway (40). These interesting data pose an intriguing question regarding the exact role of O-GlcNAc in autophagy.

In sum, our work elucidates that the activity of O-GlcNAc responds to cellular cues. We believe that there is not a paucity but an abundance of O-GlcNAc modifications during vastly dynamic biological processes, in particular in the case of HPV infection. New O-GlcNAc sites might be revealed on the previously known OGT substrates, and these could adjust biological systems to adapt to changing external and internal conditions.

## Experimental procedures

### Plasmids, cell culture, and antibodies

Cells were grown in Dulbecco's modified Eagle's medium containing 10% fetal bovine serum and 1% penicillin–streptomycin at 37 °C in a 5% CO_2_ incubator. Plasmids were as follows: ULK1 plasmids were gifts from Dr Ying Zhao. OGT plasmids were as described previously (41); ULK1-S2A (S409AS410A), 2TA (T635AT754A), and S423A plasmids were generated using specific primers (sequences available upon request) following the manufacturer's instructions (QuickChange II; Stratagene).

#### Chemicals

TMG treatment was at 5 μM for 24 h; 5S-G (OGTi) at 100 μM (prepared at 50 mM in dimethyl sulfoxide) for 24 h; CQ (100 nM for 3 h); TMG plus Glu (TMG + Glu) (23) treatment was TMG (5 μM) for 24 h and 30 mM Glu for 3 h.

#### Antibodies

ULK1 (CST; catalog no.: 8054), STX17 (Sigma; catalog no.: HPA001204), LAMP-2 (GeneTex; catalog no.: GTX103214), and LC-3 (CST, catalog no.: 2775). Rabbit anti-ULK1-pS423 antibodies were manufactured using the sequence GPFSpSSRC by Dia-An Biotech, Inc.

### HNSCC UMSCC17B cell transfection

Human HNSCC UMSCC17B cells were infected with lentiviruses expressing HPV16 E6, E7, E6/E7, or empty vectors and selected with 1 μg/ml puromycin for 2 weeks (10). The survival clones were combined.

### IP and immunoblotting assays

IP and immunobloting experiments were performed as previously described (42). O-GlcNAc was enriched by treating the cells with Glu plus TMG as previously described (treated with 5 μM TMG for 24 h and 30 mM Glu for 3 h) (41). Antibodies used were as follows: anti-ULK1 (CST; catalog no.: 8054), anti-OGT (Santa Cruz; catalog no.: sc-32921), anti-O-GlcNAc (RL2) (Abcam; catalog no.: ab2739), anti-O-GlcNAc (CTD110.6) (Sigma; catalog no.: O7764), LC3B (CST; catalog no.: 2775S), and anti-STX17 (Merck; catalog no.: HPA001204).

Peroxidase-conjugated secondary antibodies were from Jackson ImmunoResearch. Blotted proteins were visualized using the ECL detection system (Amersham). Signals were detected by a LAS-4000 (Fujifilm) and quantitatively analyzed by densitometry using the Multi Gauge software (Fujifilm). All Western blots were repeated at least three times. Silver staining analysis was performed as described (41).

### LC–MS/MS analysis of O-GlcNAc sites on ULK1

In-gel digestion was performed as previously described (25). Briefly, the ULK1 protein was resolved by SDS-PAGE, and the corresponding band was excised and cut into pieces, which were then destained, dehydrated, reduced, alkylated, and again dehydrated. The resulting gel pieces were rehydrated with a trypsin solution and incubated at 37 °C for 16 h. Peptides were extracted by 50% acetonitrile in a 5% trifluoroacetic acid buffer. The extracted peptides were dried and then analyzed by Orbitrap Elite mass spectrometer (Thermo Fisher Scientific) coupled with an EASY-nLC 1000 LC system. The mass spectrometer was performed in a data-dependent mode (top 10), and the fragmentation mode was ETD. The ETD activation times were set at 150 ms. Supplemental activation for ETD was enabled. The obtained MS raw file was processed by MaxQuant 1.6.1.0 (Max Planck Institute of Biochemistry) (43) integrated with Andromeda search engine against the UniProt Human Reference Proteome (21,006 entries, downloaded on April 2016). Cysteine carbamidomethylation was set as a fixed modification, whereas methionine oxidation, protein N-terminal acetylation, and O-GlcNAcylation on serine/threonine were set as variable modifications. The enzyme specificity was set as trypsin, and a maximum of two missed cleavage was allowed. Default parameters for the instrument were used: the initial mass tolerance for precursors was 20 ppm, the final mass tolerance for precursors was 6 ppm, and the mass tolerance for ETD ion trap fragments was 0.5 Da. For the identification of O-GlcNAcylated peptides, default parameters of MaxQuant were also used, including Andromeda score >40, delta score >6, 1% peptide-to-spectrum match-level false discovery rate, and 1% site-level false discovery rate. O-GlcNAcylation sites could be unambiguously assigned when their localization probability was higher than 0.75.

## Data availability

The MS proteomics data have been deposited to the ProteomeXchange Consortium *via* the PRIDE (44) partner repository with the dataset identifier PXD031095. The spectrum in Figure 2*D* was selected from the best PEP scan number according to MaxQuant results.

## Conflict of interest

The authors declare that they have no conflicts of interest with the contents of this article.
